# Evaluation of fertilization capability of frozen-thawed completely immotile spermatozoa collected from a white bengal tiger after interspecific ICSI with bovine oocytes

**DOI:** 10.1590/1984-3143-AR2022-0034

**Published:** 2022-07-04

**Authors:** Hai-Jun Liu, Jing-Hua Ma, Ru-Ming Liu, Zhang-Guo Liu, Hai-Jun Huang, Jian-Qiang Zou, Jian-Xun Liu, Xian-Fu Zhang

**Affiliations:** 1 College of Animal Science and Technology, Zhejiang Agriculture & Forestry University, Hangzhou, Zhejiang, China; 2 Hangzhou Safari Park, Hangzhou, Zhejiang, China; 3 College of Life Sciences, Nankai University, Tianjin, China

**Keywords:** White Bengal Tiger, cryopreservation, immotile spermatozoa, interspecific ICSI, bovine oocytes

## Abstract

The objective of this study was to evaluate the fertilization capability of White Bengal Tiger frozen-thawed completely immotile spermatozoa after interspecific intracytoplasmic sperm injection (ICSI) with bovine oocytes. The fertilization status of presumptive zygotes was assessed 18 h after ICSI by immunofluorescence staining and confocal microscopy. The fertilization rate was 34.8% (8/23), as confirmed by the extrusion of two polar bodies, or male and female pronuclei formation. For unfertilized oocytes (65.2%, 15/23), one activated oocyte had an activated spermatozoon but most were unactivated oocytes with unactivated spermatozoa (1/15, 6.7% vs 10/15, 66.7%, respectively, *p* < 0.05). These results showed that White Bengal Tiger frozen-thawed completely immotile spermatozoa retained the capacity to fertilize bovine oocytes after interspecific ICSI. This is the first report of in vitro produced zygotes using tiger immotile sperm with bovine oocytes by interspecific ICSI technique, which provides an efficient and feasible method for preservation and utilization of endangered feline animals.

## Introduction

As one of the established assisted reproductive technologies, intracytoplasmic sperm injection (ICSI) has improved fertilization capability, in particular when the quality of semen is poor. Live offspring were produced by ICSI using immotile sperm from cattle (Goto et al., 1990b) and humans (Geber et al., 2012; Nordhoff et al., 2013). Viable spermatozoa selected by laser from frozen-thawed immotile spermatozoa resulted in similar fertilization and cleavage rates compared with their fresh counterparts after ICSI (Chen et al., 2017).

Low semen quality with high incidence of morphologically abnormal spermatozoa is observed in several wild felids such as the cheetah (*Acinonyx jubatus*) and leopard (*Panthera pardus*). Past work showed that interspecific ICSI using cheetah and leopard spermatozoa with domestic cat oocytes, embryos reached a high blastocyst rate (32.6%; 21%, respectively) (Moro et al., 2014). These results suggest that ICSI is a promising technique to assist felid reproduction. Furthermore, in vitro-derived lion embryos were produced following oocyte vitrification and ICSI (Zahmel et al., 2021).

Buarpung et al. (2013) demonstrated that domestic cat spermatozoa derived from frozen-thawed testicular tissues retained their fertilizing ability and could be used to produce ICSI-derived embryos.

As early as 1990, live-born tiger offspring have been successfully produced through transferring in vitro fertilization (IVF)-derived embryos (Donoghue et al., 1990). However, there has been little further research carried out for tiger species (Donoghue et al., 1992; Wayan Kurniani Karja et al., 2016).

To date, the fertilization competence of tiger sperm as well as immotile sperm in endangered feline by ICSI has not been examined.

The objective of this study was to investigate the fertilization capability of cryopreserved immotile sperm collected from a White Bengal Tiger (*Panthera tigris tigris*) after interspecific ICSI with bovine oocytes.

## Methods

### Ethics statement

All experiments involving animals were conducted under the protocol approved by the Animal Care and Use Committee of the College of Animal Science and Technology, Zhejiang Agriculture & Forestry University (No.2019/4/8).

### Chemicals

All chemicals were purchased from Sigma Chemical Co. (St. Louis, MO, USA) unless otherwise stated.

### Semen collection, cryopreservation and thawing

An adult male White Bengal Tiger kept at the Hangzhou Safari Park was used in this study. For semen collection, the male was anaesthetized with atropine and ketamine administered by blow dart. Semen was collected via electroejaculation with an electroejaculator (MDW-1, Chengdu Huazhi, Chengdu, China). The probe was lubricated and inserted approximately 20 cm into the tiger's rectum. The current was applied in a 5-sec-on/5-sec-off pattern using 3 to 6 V with a total of 50 stimuli. The collected semen was assessed for ejaculate volume, spermatozoa motility and concentration.

Semen was diluted to a concentration of 4×10^7^ sperm /ml with Optidyl extender (IMV Technologies, 61300 L'Aigle, France). Diluted semen was packaged into 0.25-ml French straws (IMV Technologies). Straws were held 3 cm above the surface of liquid nitrogen for 10 min before being plunged into liquid nitrogen. Thawing was performed by plunging a straw into a 37 °C water bath for 20 sec.

### Oocyte collection and in vitro maturation

Oocyte collection and in vitro maturation were performed as described previously (Lin et al., 2016). Bovine ovaries were collected from a local slaughterhouse. Cumulus–oocyte complexes (COCs) were recovered by aspiration. Selected COCs were placed in TCM-199 (Gibco, Grand Island, NY, USA) based maturation medium wells and cultured at 38.5 °C in a humidified atmosphere of 5% CO2 in air for 24 h.

### ICSI

After in vitro maturation, oocytes were denuded with 0.5% hyaluronidase. Those with first polar bodies and normal morphology were selected for ICSI. Thawed semen was centrifuged in Sydney IVF Sperm Medium (Cook Group, Bloomington, Indiana, USA) and diluted to a concentration of 5×10^6^ with Sydney IVF Fertilization Medium (COOK Group). The sperm suspension was then mixed with 10% (w/v) polyvinylpyrrolidone (COOK Group) at a ratio of 1:4. Each spermatozoon was aspirated tail first into an injection pipette with an inner diameter of 5-5.7μm (Origio, Cooper Surgical, Denmark). The injected oocytes were cultured in Sydney IVF Cleavage Medium (COOK Group) at 38.5 °C in a humidified atmosphere of 5% CO2 in air for 18h.

### Immunofluorescence staining and confocal microscopy

The presumptive zygotes were collected at 18 h after ICSI. The method for immunofluorescence staining was carried out as described previously (Liu and Liu, 2018). Samples were fixed in 0.3% Triton X-100 and 2% formaldehyde in PBS (Gibco), then blocked with 1% goat serum in PBS (Gibco). Samples were incubated with mouse anti-a tubulin (1:100 dilution) and fluorescein isothiocyanate (FITC)-conjugated goat antimouse immunoglobulin G (1:100 dilution) in PBS. DNA was stained with 10 μg/mL Hoechst 33342. Finally, the samples were mounted on slides with antifade kits (Invitrogen, Life Sciences, Carlsbad, CA, USA), and observed using laser scanning confocal microscopy (ZEISS LSM710, Jena, Germany). The presumptive zygotes with two polar bodies (under bright field) or two pronuclei (under fluorescence) were considered to be fertilized.

### Statistical analysis

The data were analyzed using the Chi‐squared test with GraphPad InStat 3 software, and *p*≤0.05 was considered significant.

## Results

### Semen quality assessment

After electroejaculation, collected semen was assessed for quality. The semen volume was 4.2 ml and sperm concentration was 1.7×10^7^/mL. No motile spermatozoa were found; thus, the semen motility was 0. Immotile sperm were subjected to freezing.

### The fertilization capability of cryopreserved immotile sperm

**Figure 1:**
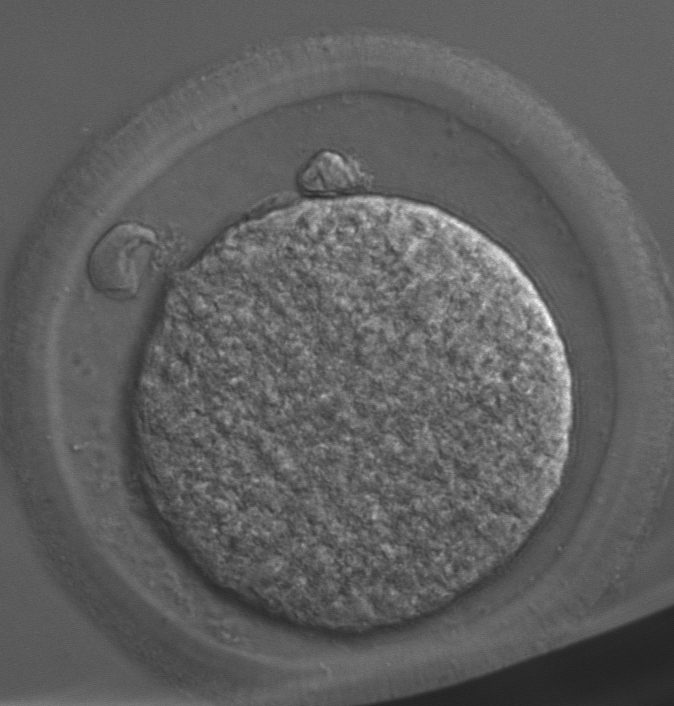
Extrusion of two polar bodies in a presumptive zygote under bright field.

**Figure 2:**
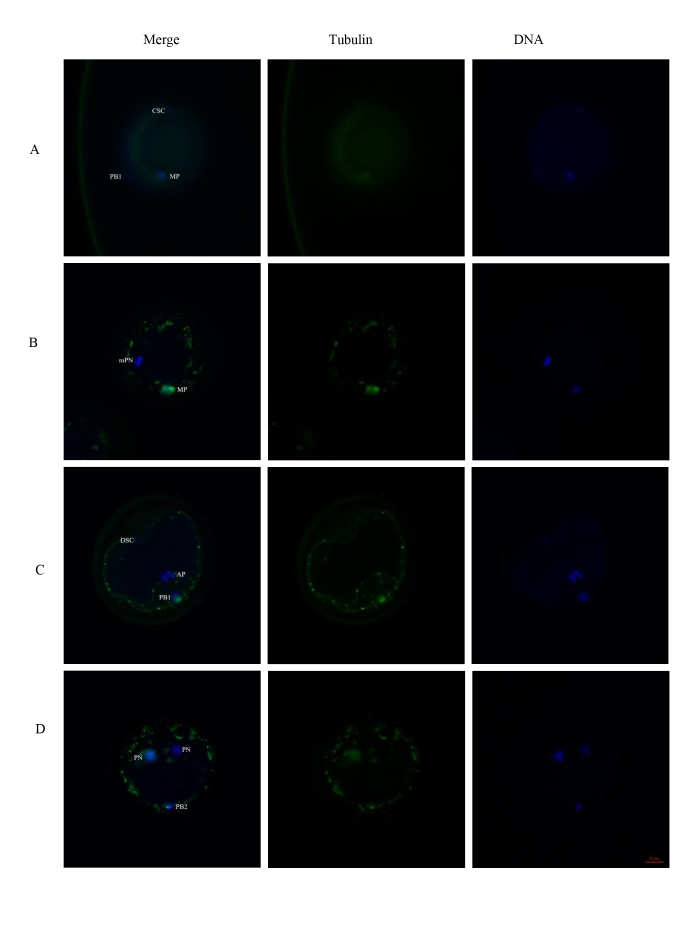
Status of microtubules and chromatin in presumptive zygotes. (A) Spermatozoa and oocytes were unactivated. Sperm chromatin kept intact. Oocyte arrested in metaphase II stage. (B) Spermatozoa was activated, but oocyte not. Male pronucleus formed. Oocyte arrested in metaphase II stage. (C) Spermatozoa and oocyte were activated mutually, Spermatozoa head turned to round and underwent nuclear decondensation. Oocyte developed further to anaphase. (D) Female and male pronuclei formed but remained separate. Notes: 1. Green, α-tubulin; Blue, DNA in confocal images. 2. CSC: condensed sperm chromatin; MP: metaphase; PB1: first polar body; mPN: male pronucleus; DSC: decondensed sperm chromatin; AP: anaphase; PN: pronucleus; PB2: second polar body. 3. Scale bar: bar=20 μm.

Eighteen hours after ICSI, 5/23 presumptive zygotes exhibited two polar bodies (Figure 1), and another three formed male and female pronuclei, as confirmed by fluorescence detection (Figure2). The total fertilization rate was 34.8% (8/23). The other 15 presumptive zygotes were defined as unfertilized (Table 1). Of these, 10/15 oocytes and associated spermatozoa remained unactivated. It was shown that sperm chromatin remained intact and oocytes were arrested at metaphase II stage. In the other presumptive zygotes, 4/15 exhibited activated sperm with male pronucleus formation, 1 but non-activated oocytes were arrested at metaphase II stage. Only 1/15 spermatozoa/oocyte were mutually activated, in which the spermatozoon head became round and underwent chromatin decondensation, and the oocyte developed to anaphase stage (Figure 2). The proportion of both unactivated oocytes and spermatozoa was significantly higher than that with an activated oocyte and activated spermatozoon (10/15, 66.7% vs 1/15, 6.7%, *p* <0 .05) (Table 2).

**Table 1:** Fertilization assessment of the presumptive zygotes.

**No. of presumptive zygotes**	**No. of two polar bodies (%)**	**No. of male and female pronuclei formation (%)**	**No. of total fertilizated (%)**	**No. of unfertilizated (%)**
23	5 (21.7)	3 (13.1)	8 (34.8)	15 (65.2)

**Table 2:** Status of microtubules and chromatin in the unfertilized oocytes.

**Unfertilized Oocytes**	**Sperm and oocytes unactivated (%)**	**Sperm activated only（%)**	**Sperm and oocytes activated mutually (%)**
15	10(66.7)^a^	4(26.6)	1(6.7)^b^

Note: Different superscripts within the same column (e.g. a, b,) represent significant differences *(p* < 0.05).

## Discussion

This is the first report of in vitro produced zygotes using tiger immotile sperm with bovine oocytes by interspecific ICSI technique, which provides an efficient and feasible method for preservation and utilization of endangered feline animals. Our results showed that White Bengal tiger completely immotile spermatozoa can be frozen and have the capacity to fertilize bovine oocytes after interspecific ICSI, reaching a fertilization rate of 34.8% (8/23).

The reason that tiger sperm can activate bovine eggs is closely related to fertilization mechanisms. At fertilization, the sperm releases an oocyte activating factor(s) (OAF) to activate oocytes. Parrington et al. (1996) reported that the OAF is a 33 kDa protein localized in the equatorial segment of sperm head in hamster. The sperm-borne activation factor(s) is not strictly species-specific, this point was evidenced by activated mouse oocytes through injecting sperm from different species such as sea urchin, human, hamster, rabbit, pig or fish (Wakayama et al., 1997; Kimura et al., 1998). Recently, Gambini et al. (2020) reported that injection of Zebra or horse sperm activated porcine oocytes.

Moro et al. (2014) performed interspecific ICSI using cheetah and leopard spermatozoa with domestic cat oocytes. Different from our study in spermatozoa source, they used frozen-thawed motile spermatozoa, not cryopreserved completely immotile spermatozoa.

In this study, both unactivated oocytes and spermatozoa composed of the main proportion of unfertilized oocytes (10/15, 66.7%). This result showed that these injected spermatozoa losed their capability to activate oocytes. We presumed that ejaculated immotile sperm were more vulnerable to frozen-thawed procedure. Similiar to the current study, in domestic cat, Buarpung et al. (2013) demonstrated that both unactivated oocytes and spermatozoa accounted for the largest proportion (35/77, 45.4%) of all injected oocytes at 18 h after ICSI with frozen testicular spermatozoa.

For human, viable spermatozoa identified by laser, no significant differences were found in fertilization rates after ICSI using fresh and frozen immotile spermatozoa (75.00% vs 81.48%, respectively) from ejaculate sources (Chen et al., 2017). For the current study, we predict that the fertilization rate could be improved by the selection of viable sperm from immotile sperm before ICSI.

For normal bovine zygotes (with two pronuclei) that resulted in blastocysts, the time-points from IVF to pronuclei appearance and fading were 10.4 h and 25.5 h, respectively (Suzuki et al., 2021). In the present study, 8 of 23 zygotes formed male and female pronuclei 18 h after ICSI, within the expected period of pronuclear appearance to fading (10.4~25.5h).

## Conclusion

Frozen-thawed White Bengal tiger completely immotile spermatozoa have the capacity to fertilize bovine oocytes after interspecific ICSI. This result provides a new perspective for preserving endanged mammals by assisted reproductive technologies.
